# Tick phenology, tick-host associations, and tick-borne pathogen surveillance in a recreational forest of East Texas, USA

**DOI:** 10.1371/journal.pone.0330826

**Published:** 2025-08-21

**Authors:** Jordan Salomon, Haydee Montemayor, Cassandra Durden, Dorcas Abiara, Rachel E. Busselman, Gabriel L. Hamer, Sarah A. Hamer

**Affiliations:** 1 Ecology and Evolutionary Biology Program, Texas A&M University, College Station, Texas, United States of America; 2 Department of Veterinary Integrative Biosciences, Texas A&M University, College Station, Texas, United States of America; 3 Department of Entomology, Texas A&M University, College Station, Texas, United States of America; University of Minnesota, UNITED STATES OF AMERICA

## Abstract

Management of tick-borne disease necessitates an understanding of tick phenology, tick-host associations, and pathogen dynamics. In a recreational hotspot outside of one of the largest cities in the United States, we conducted a year of monthly standardized tick drag sampling and wildlife trapping in Sam Houston National Forest, a high use recreation site near Houston in east Texas, US. By sampling 150 wildlife hosts of 18 species, including rodents, meso-mammals, deer, reptiles, and amphibians, we collected 87 blood samples, 90 ear biopsies, and 861 ticks representing four species (*Amblyomma americanum, Dermacentor variabilis*, *Ixodes scapularis* and *Ixodes texanus*). Drag sampling yielded 1,651 questing ticks of three species: *A. americanum* (921)*, D. variabilis* (10), and *I. scapularis* (720). Off-host larval *A. americanum* abundance peaked in July, followed by peak infestations of wildlife, predominantly raccoons, in August. Off-host *I. scapularis* larvae abundance peaked in spring (March-May), while very few were removed from hosts and only a single *I. scapularis* nymph was found throughout the study via dragging in June. In contrast, both off-host and on-host adult *I. scapularis* occurred most frequently in the winter. Overall, tick infections included 25.3% (183/725) with *Rickettsia buchneri*, 15.5% (112/725) *Rickettsia amblyommatis*, 8.0% (58/725) *Rickettsia tillamookensis,* 0.8% (6/725) *Rickettsia* spp., and a single tick with a hard tick relapsing fever *Borrelia* spp.; no tick tested positive for *Borrelia burgdorferi*. Characterizing tick phenology, tick-host associations, and tick-borne bacteria fills important knowledge gaps for the risk of tick-borne diseases in pine-dominated forests of this region.

## Introduction

Tick-borne pathogens continue to emerge resulting in an increased reports of morbidity and mortality as well as a significant economic burden [1]. Reduction of human disease can be achieved by preventing vector bites, reducing enzootic transmission of pathogens, or interrupting pathogen spillover from sylvatic to anthropic cycles. To mitigate tick-borne disease risk, several ecological details can provide key information, including the seasonality of host-seeking behavior of different tick species and life stages (i.e., phenology), frequency of vertebrate host/vector encounters (i.e., host associations), and pathogen occurrence. This ecological understanding is surprisingly poorly understudied, leading to inadequate mitigation and the surge in human tick-borne diseases in the United States (US) [2]; this is especially true in the southern US where Lyme disease is rare yet other tick-borne diseases prevail.

Tick phenology, or the seasonal peak abundance of successional host-seeking (i.e., off-host) and then host-feeding (i.e., on-host), directly impacts tick-borne pathogen transmission through several mechanisms. For some pathogens, transmission can increase when there is synchronous overlap across vector life stages [3,4], direct co-feeding on the same host [5,6], or peak phenology of nymphs or adults followed by larvae from the next generation [7]. Furthermore, variation in phenology exists among tick species [8,9], and within the same species across different regions [10,11]. This inter- and intra-species variation in tick phenology regulates tick-borne pathogen prevalence among sylvatic cycles. Given peak phenology of infected life stages determines the risk of human exposure to ticks and resulting patterns of disease in some systems [12], tick seasonal activity is critical for public health messaging.

The simultaneous study of both on- and off-host ticks using standardized surveillance approaches allows for pathogen detection and understanding transmission dynamics. Knowledge of tick-host associations can be useful for understanding patterns of tick infection, identifying which hosts could be most effective for targeted-host interventions, and which hosts could disperse ticks and tick-borne pathogens into new ranges [13]. Furthermore, they can identify smaller scale community level dynamics (i.e., host community abundance, host diversity, trophic level diversity, etc.) that influence the phenology of vectors and the pathogens they transmit [14,15]. However, longitudinal field surveillance of wildlife is resource and labor intensive, and thus not as commonly conducted as active surveillance for host-seeking ticks or passive surveillance with publicly submitted ticks.

Although Lyme disease is the number one reported tick-borne disease in the US [16], it is rarely acquired in the Texas and the southern US, where spotted fever group rickettsioses are more common. Caused by a suite of different obligate intracellular bacteria in the *Rickettsia* genus, each species has different wildlife host-associations and ecologies [17,18]. Some of the most common causes of tick-borne rickettsioses in the US are *Rickettsia rickettsii* and *Rickettsia parkeri* transmitted by *Amblyomma americanum* (Ixodida: Ixodidae Linnaeus, 1758) and *Dermacentor variabilis* (Ixodida: Ixodidae Say, 1812). More recently, several other *Rickettsia* species have been found in high abundance throughout the US and may be pathogenic (i.e., *Rickettsia amblyommatis* and *Rickettsia tillamookensis*) [19–22].

Despite some characterization of pathogens in ticks removed from humans across Texas [23,24], ecological studies of ticks in the region remain rare, in part because fewer human cases of tick-borne diseases are reported relative to other regions in the country. In this study we describe the ecology of sylvatic ticks and tick-borne bacteria in a national forest of East Texas (TX), US, with high human activity over the course of a year. Because it is known in other regions that juvenile *I. scapularis* are associated with small rodents and herpetofauna, while adults are associated with deer; we designed this survey targeting these hosts. With a standardized sampling regime our objectives were to report: 1) phenology of host-seeking (i.e., off-hosts) and host-feeding ticks (i.e., on-hosts), 2) tick-host associations, and 3) and tick-borne pathogen occurrence in ticks and their wildlife hosts.

## Materials and methods

### Field site description

**Fig 1:**
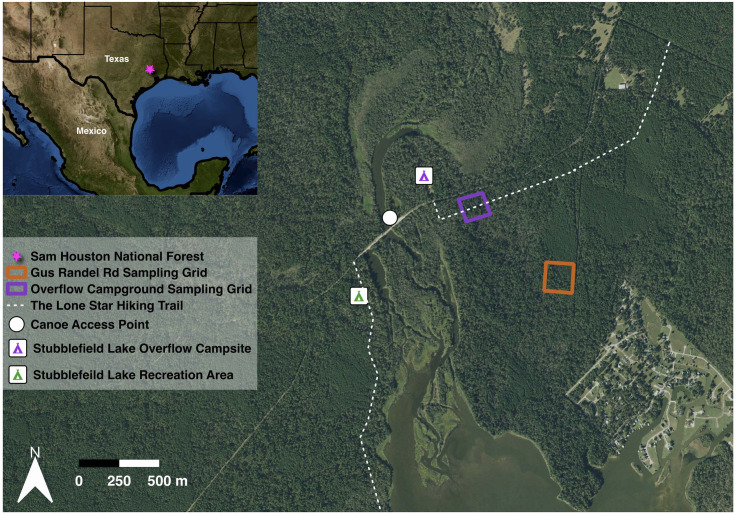
Map of the location of the sampling grids from May 2021 to May 2022 within a region of high human activity of Sam Houston National Forest, TX, USA. The two sampling grids are indicated by the orange and purple squares. One inset map shows the context of the study sites in east TX, US. Map was created in QGIS with USGS National Map Imagery (public domain data provided by the U.S. Geological Survey, accessed June 18^th^, 2025) for the base map.

This study was conducted at Sam Houston National Forest in East Central TX, US (Fig 1), as described in a previous study [25]. Briefly, vegetation is comprised of loblolly pines (*Pinus taeda*), shortleaf pines (*Pinus echinata*), oak (*Quercus* spp.), elm (*Ulmus* spp.), and sweetgum (*Liquidambar* spp.), dwarf palmetto (*Sabal minor*), yaupon holly (*Ilex vomitoria*), and American beautyberry (*Callicarpa americana*). Collection grids were established in forested compartments that do not receive prescribed burns, as previous research indicates that *A. americanum, D. variabilis,* and *I. scapularis* ticks can be found questing in higher abundances in unburned areas of Sam Houston National Forest [26]. Sam Houston National Forest has numerous anthropogenic activities, as the forest is used for logging, livestock agriculture, recreation (e.g., camping, hiking, boating, hunting), and has some residential neighborhoods nested within the forest (Fig 1).

### Sampling interval

Two sampling grids of 10,000 m^2^ each were composed of 100 m six parallel lines (running South to North) intersected by six parallel lines (running East to West) every 20 m resulting in a total of 20,000 m^2^ sampled for wildlife. Vegetation was pruned to allow personnel to walk the transects throughout the year for tick dragging and wildlife trapping. One sampling grid was near a campground intersected by the popular Lone Star Hiking Trail, while the second grid is approximately half a kilometer away (Fig 1). Both grids were sampled monthly for a single night each starting in June 2021 and ending May 2022 for a standardized effort to analyze phenology; this involved setting all traps at the first grid for one night, followed by movement of all traps to the second grid for one night. Outside this standardized monthly sampling, additional host sampling efforts occurred to increase the sample sizes for tick-host association analyses and tick-borne pathogen surveillance (see below) but were not included in any phenology analyses. These additional sampling efforts included one additional trap night in May 2021 before the standardized sampling beginning in June 2021 targeting rodents and meso-mammals; and then four additional rodent trap nights in April and May of 2022 to capitalize on the spring boom of rodent populations. During these additional trapping efforts on the routine sampling grids, we also included herpetofauna and human tick collections. Lastly, on the first two opening weekends of deer hunting season in the national forest in November 2021, we sampled harvested *Odocoileus virginianus* (white tailed deer) at the eastern ranger station.

### Mammal live trapping

Live trapping protocols to target a diversity of known key hosts of ticks (i.e., rodents and meso-mammals) are previously described and were approved by the Texas A&M University Institutional Animal Care and Use Committee (IACUC 2021−0124 D C) [25]. Briefly, 111 traps were set for one night at the first grid followed by one night at the second grid for standardized monthly sampling. Each of 36 trapping stations had a two small Sherman live traps (H.B. Sherman Traps, Tallahassee, FL, US) facing opposite direction of each other, baited with sunflower seeds. Additionally, 24 live traps (Tomahawk Live Trap, Hazelhurst, WI, US), and 15 extra-large Sherman live traps were placed around fallen logs or game trails near a trapping station to increase likelihood of trap success; these were baited with combinations to peanut butter and wet cat food on tortillas. No pre-baiting occurred for any trap type. For phenological assessments of on-host ticks a total of 222 trap nights were included for each month (111 at each grid).

Each captured animal was brought to a central processing location for anesthesia [27] and sampling. All animals were released upon recovery from anesthesia at the site of capture. Metadata were recorded for each capture (i.e., species, weight, sex, age, and reproductive status), and each animal was given a metal ear tag with a unique number. Samples removed from each capture included: ectoparasites, a 2 mm ear punch biopsy, and a blood sample. Tick checks of the entire body were conducted with special attention to the head, a common attachment site for ticks [28,29]. All collected ecto-parasites and ear biopsies were stored in 70% ethanol and once separated into clot and serum, blood was stored at −20 °C until further processing.

### Herpetofauna sampling

During sampling events, herpetofauna were opportunistically caught by hand and checked for ticks. Species, location, and the number of ticks removed into 70% ethanol with fine tipped forceps, was recorded. To openly share occurrence data photos of reptiles and amphibians were submitted to iNaturalist (www.inaturalist.org/).

### Off-host tick sampling from vegetation

Monthly tick drag sampling was conducted at both sampling grids following a standardized protocol [30]. All 12 transects (6 parallel transects from each grid) were sampled totaling 1,200 m^2^ sampled per month. Ticks removed from project personnel during the sampling events were also saved and further processed in the lab.

### Tick identification

All ticks were identified to species, life stage, and sex with a dissecting microscope [31,32]. Representative ticks of each life stage and sex were submitted as voucher specimens to the Texas A&M University Insect Collection of the Department of Entomology (Accession number TAMUIC768), with collection information of these voucher specimens also submitted to the Global Biodiversity Information Facility data source. Engorgement status of on-host ticks on a scale of zero to five was assigned (zero = flat ticks; five = engorged and presumed to be near repletion) [33]. Total off-host tick abundance for all transects on both grids was divided by 1,200 m^2^ and multiplied by 100 m^2^ to calculate density for each species and life stage per 100 m^2^. Similarly, on-host tick abundance, or the number of ticks removed per animal per capture [34], was quantified and averaged across host species and for each month, with standard error of the means. All tick occurrences were submitted to the Center for Disease Control and Prevention ArboNET Tick Module.

### DNA extractions of blood clot, ear punch biopsies, and ticks

While all host tissues were processed molecularly, a stratified subset of ticks was as selected for individual molecular analysis due to limited resources. This included all collected off-host adults and nymphs, a maximum of 20 off-host larvae randomly selected from each ‘larval bomb’, all ticks from personnel, and a maximum of ten on-host ticks per species and life stage from each individual animal, stratified by engorgement score when possible. Ear punch biopsies, blood clots, and tick DNA were extracted with a commercial DNA extraction kit (E.Z.N.A Tissue DNA Kit; Omega Bio-Tek, Norcross, GA, US) as described previously [25].

### PCR for identifying rodent, *Rickettsia*, and *Borrelia* species

Mice and rats were molecularly identified to verify field identifications by targeting the cytochrome B (*Cytb*) gene [25,35].

To test for the presence of *Rickettsia* spp. within hosts (i.e., testing blood clot and ear punch biopsies) and a subset of ticks (both on-host and off-host), we targeted three different genes: outer membrane protein A (*ompA*), outer membrane protein B (*ompB*), and citrate synthase (*gltA*) by conventional PCR [33,36,37]. All *Rickettsia* PCR protocols used FailSafe™ (Lucigen, Middleton, WI, US) reagents and included PCR grade water as a negative control, extraction negatives, and used a field collected *R. parkeri* as a positive control [25]. Samples were considered positive only if the PCR product was successfully sequenced. Some vertebrate hosts were previously assayed for *Rickettsia* infection in our study of fleas and flea-borne *Rickettsia* [18] leading to the detection of tick-borne *Rickettsia* (*R. amblyommatis*); those results are again reported herein.

To test for the presence of *Borrelia* spp. in hosts and ticks, we ran both a multiplex qPCR targeting 16S rRNA [38] and a nested PCR [25] targeting the intergenetic spacer region between 16S and 23S rRNA. Every PCR reaction included a negative control of PCR-grade water, the extraction negatives, and a positive control from field collected samples of *B. burgdorferi* sensu stricto. Additionally, *Borrelia lonestari* was a positive control for relapsing fever group in the qPCR assay. Samples with CT < 34 were considered positive on the qPCR assay and were considered positive on the nested assay only if the product was successfully sequenced. Pathogen prevalence was calculated as the number of sequence-positive individual samples divided by number of individual samples tested.

### DNA sequencing

All conventional PCR products were visualized via gel electrophoresis and amplicons were purified with ExoSAP-IT™ (Affymetrix, Santa Clara, CA, US). Sanger sequencing was performed (Eton Bioscience Inc, San Diego, CA, US). Sequences were trimmed and sequences with high quality chromatographs were compared to published sequences in NCBI GenBank (Geneious v 9.1.8).

For host species identification, all sequences were >99% identical to published sequences of NCBI GenBank. We required >97% sequence homology to a published sequence to identify bacteria to species level. In the instance where a sample was > 95 but <97% identical to published sequences, we assigned the identity only to the genus level. Co-infections were determined when a different species of *Rickettsia* was identified using different assays. Sequences were submitted to NCBI GenBank (PQ163307-PQ163452, PQ083791-PQ083830, PQ038313-PQ0383229, PQ247227-PQ247467, and PQ213429).

## Results

### Host inventory

We conducted a total of 7,946 trap nights and captured 97 rodents and meso-mammals comprised of 61 unique individuals, with an overall trapping success of 1.2%. We captured seven different mammalian species including *Ochrotomys nuttalli* (golden mouse; n = 32)*, Peromyscus leucopus* (white-footed mouse; n = 17)*, Neotoma floridana* (eastern woodrat; n = 5)*, Glaucomys volans* (southern flying squirrel; n = 2)*, Sciurus carolinensis* (eastern gray squirrel; n = 1)*, Didelphis virginiana* (Virginia opossum = 29), and *Procyon lotor* (raccoon; n = 11). We had many recaptured mammals over the year. Among the 49 mouse (*O. nuttalli* and *P. leucopus*) captures 24 were unique individuals, of 29 opossum captures 20 were unique individuals, and of the 5 woodrat captures 3 were unique individuals. Of the recaptures, the highest frequency per species was two *N. floridana* captured in two different months, one *P. leucopus* caught in four different months, two *O. nuttalli* were each caught in four different months, and one *D. virginianus* was caught in five different months. We captured 43 reptile or amphibian individuals including *Incilius valliceps* (Gulf Coast toad; n = 22), *Scincella lateralis* (little brown skink; n = eight), *Storeria dekayi* (Dekays brown snake; n = three), *Hyla cinerea* (green tree frog; n = three), *Terrapene carolina triunguis* (three-toed box turtle, n = two), *Anaxyrus woodhousii* (a Woodhouse’s toad, n = one), *Anaxyrus fowleri* (Fowler’s toad, n = one), *Pseudacris crucifer* (spring peeper, n = one), *Anolis carolinensis* (green anole, n = one), and *Pantherophis obsoletus* (western rat snake; n = one).

### Tick-borne bacteria detection in hosts

Of 87 blood clot samples and 90 ear biopsies, no hosts were positive for *Borrelia* species. As previously detected in our study of fleas and flea-borne *Rickettsia* [18], four *P. lotor* and one *O. nuttalli* were positive for *Rickettsia amblyommatis* (Table 1; Accession numbers PP119085, 87–89). All five hosts positive for *R. amblyommatis* had ticks at the time that they tested positive, with the highest abundance of 86 ticks on a raccoon in September (Table 1).

**Table 1:** Attributes of the five mammalian hosts that tested positive for *R. amblyommatis* (as previously reported in our study of fleas and flea-borne *Rickettsia*; [18]) and ticks present at the time of host positivity, east Texas.

Time of positivity	PCR assay of positivity	Host species	Ticks on-host	*Rickettsia* prevalence of on-host ticks	*Rickettsia* species in on-host ticks	Host recapture notes
August 2021	*ompA* blood clot	*Procyon lotor*	50 *A. americanum* L, 12 *A. americanum* N, 2 *D. variabilis* A females	30.0% (3/10)	*R. amblyommatis*	
September 2021	*ompA* ear biopsy	*Procyon lotor*	85 *A. americanum* L, 1 *A. americanum* N	18.2% (3/11)	*R. amblyommatis*	Caught first in May 2021, had 7 ticks attached at that time (2 *D. variabilis* females, 5 males.) Host and all the ticks were negative.
September 2021	*gltA* ear biopsy	*Procyon lotor*	15 *A. americanum* L 7 *A. americanum* N, 1 *D. variabilis* A females, 2 *I. texanus* L, 1 *I. texanus* N	50.0% (5/10)	*R. amblyommatis*	
April 2022	*gltA* ear biopsy	*Procyon lotor*	2 *A. americanum* N, 1 *D. variabilis* A male	33.3% (1/3)	*R. amblyommatis*	
May 2022	*gltA* ear biopsy	*Ochrotomys nuttalli*	2 *I. scapularis* L	100 (2/2)	*R. tillamookensis, R. buchneri*	Caught first in April 2022 with no ticks and host was negative.

USA. L = larvae, N = nymphal, and A = adult. For hosts that were recaptures, capture history is included.

### Tick inventory

Overall, 2,549 ticks were collected from vegetation, wildlife hosts, or project personnel between May 2021 to May 2022: 1,651 from vegetation, 434 from wildlife trapped on the two sampling grids, 427 from ten deer, and 37 from personnel during sampling events throughout the year. *Amblyomma americanum* was the most abundant (1,349) followed by *I. scapularis* (945)*, D. variabilis* (163)*, I. texanus* (81), and 11 ticks were morphologically unidentifiable. A total of 725 from the collected 2,549 ticks were further processed for molecular detection of tick-borne bacteria (Table 2).

**Table 2:** Results of tick testing for *Rickettsia* and *Borrelia* species, May 2021 to May 2022, east Texas, USA from both on and off-hosts.

Tick-borne bacterial species	*A. americanum*			*D. variabilis*			*I. scapularis*			*I. texanus*			unidentifiable			Total positives
	L	N	A	L	N	A	L	N	A	L	N	A	L	N	A	
*Rickettsia amblyommatis*	18 (20)	60 (42.6)	21 (51.2)	1(2.1)	1 (11.1)	2 (2.0)	4 (3.2)	0	3 (2.2)	0	0	2 (12.5)	0	0	0	112
*Rickettsia buchneri*	1 (1.1)	1 (0.7)	4 (9.8)	3 (6.25)	0	0	79 (62.7)	1 (100)	63 (47.0)	0	0	0	0	0	0	183
*R. tillamookensis*	0	0	0	0	0	0	5 (4.0)	0	22 (16.4)	0	0	0	0	0	0	27
Uncultured *Rickettsia* spp.	0	0	0	0	0	0	0	0	1 (0.6)	0	0	0	0	0	0	1
*Rickettsia* spp.	0	0	0	0	0	0	0	0	5 (3.7)	0	0	0	0	0	0	5
Co-infection *R. tillamookensis* & *Rickettsia buchneri*	0	0	0	0	0	0	23 (18.3)	0	8 (6.0)	0	0	0	0	0	0	31
Co-infection *R. amblyommatis* & *Rickettsia buchneri*	0	0	0	0	0	0	0	0	1 (0.8)	0	0	0	0	0	0	1
Hard tick relapsing fever *Borrelia* sp.	0	0	0	0	0	1 (1)	0	0	0	0	0	0	0	0	0	1
Abundance of ticks tested (N = 725)	90	141	41	48	9	102	126	1	134	4	12	16	1	0	0	
Abundance of ticks collected (N = 2,538)	1142	165	42	51	9	103	718	1	226	6	19	56	1	1	9	

Reported are the number of ticks that tested positive, with the prevalence in parentheses. L = larvae, N = nymphal, and A = adult.

### Tick-borne pathogen detection in ticks both on and off-hosts

A total of 725 ticks from the year of collection were extracted and tested for *Borrelia* and *Rickettsia* species (Table 2). We detected three different *Rickettsia* spp. (*R. amblyommatis, R. tillamookensis,* and *R. buchneri*) and one hard tick relapsing fever *Borrelia* sp. (Table 2). Additionally, we identified five ticks that met our criteria to be called positive for *Rickettsia*, but not to species (Table 2). Lastly, one *Rickettsia* spp. -positive *I. scapularis* matched 99.72% equally identical to several sequences determined as *Rickettsia raoultii* isolated from Kazakhstan (MW430400.1), *Rickettsia conorii* subsp. *raoultii* China (MN450401.2), and uncultured *Rickettsia* spp. from Hungary (LC060713.1). Identification of *R. buchneri* was predominantly based on *ompA* sequences, which shared 99.76% homology with multiple Genbank entries, including several named as *Rickettsia* endosymbiont of *I. scapularis* (REIS) and the genome of *Rickettsia tamurae* subsp. Buchneri (Genbank accession no, CP113531 [39]). Based on the *gltA* sequence, 57 of the 183 positive *Rickettsia buchneri* ticks had 100% match to multiple Genbank entries, including *R. monacensis* (LC507602.1, MH618388.1, and KX987342.1), uncultured *Rickettsia*, REIS, and the same genome of *Rickettsia tamurae* subsp. Buchneri as was matched for *ompA*. We refer to these sequences as *R. buchneri* given it is the newer name for REIS [40]. No ticks were positive for *B. burgdorferi*, but one adult male *D. variabilis* removed from an opossum in June was positive for a hard tick relapsing fever *Borrelia* species. This sample was negative on the qPCR but positive several times on the IGS 16S-23S nested PCR. After the initial PCR screening, the sample was subsequently run on the nested assay in triplicate of each “neat” DNA and at a 1:10 dilution, both of which produced a clean sequence that again had a 100% identical match to *Borrelia miyamotoi* isolated from Japan (AP024371.1). All extraction negatives and negative controls were negative; no *Borrelia miyamotoi* positive control was used in these assays.

### Tick-borne pathogen detection in ticks removed from rodents, meso-mammals, and herpetofauna sampling

Following the molecular strategy previously mentioned, we selected 10 ticks of each species from each host among the 434 ticks removed from wildlife trapped on the grids. This resulted in 234 ticks tested for pathogens consisting of 46 *A. americanum* (28 larvae and 18 nymphs), 145 *D. variabilis* (48 larva, eight nymphs, and 89 adults), 11 *I. scapularis* (six larvae and five adults), and 32 *I. texanus* (four larvae, 12 nymphs, and 16 adults). We detected *R. amblyommatis* in 20 *A. americanum* (43.5%), three *D. variabilis* (2.1%), one *I. scapularis* (9.1%), and two *I. texanus* (6.3%). *Rickettsia buchneri* was detected in three *D. variabilis* (2.1%) and six *I. scapularis* (54.5%). Two *I. scapularis* were co-infected with *R. tillamookensis* and *Rickettsia buchneri* (18.2%).

### Pathogen detection in ticks removed deer

Again, following the testing regime of 10 ticks per species per host, among the 427 ticks removed from 10 deer, a total of 154 ticks comprised of 32 *A. americanum* (19 larva, 12 nymphs, one adult) and 122 *Ixodes scapularis* (four larvae, 118 adults). The most abundant bacterium was *Rickettsia buchneri* which was detected in 54 *I. scapularis* (44.3%) and three *A. americanum* (9.4%). The second most abundant bacterium in ticks removed from deer was *R. tillamookensis*, with 28 positive *I. scapularis* (23.0%). Furthermore, these deer collected ticks were positive for *R. amblyommatis* where nine were *A. americanum* (28.1%) and five were *I. scapularis* (4.1%). We identified five *I. scapularis* (4.1%) to be co-infected with *R. tillamookensis* and *Rickettsia buchneri* and one *I.* scapularis (0.8%) co-infected with *R. amblyommatis* and *Rickettsia buchneri*. Six of the positive ticks removed from deer could only be identified to the genus *Rickettsia,* all of which were *I. scapularis* (5.0%). All ten deer hosted these positive ticks; however, the deer with the highest tick burden (219) also had the most positive ticks (21).

### Pathogen detection in ticks removed from personnel conducting the field work

All thirty-seven ticks collected from humans during sampling events (either attached or crawling on humans) were tested for tick-borne pathogens. Thirty-three of these ticks were *A. americanum* (one larva, 12 nymphs, 20 adults) and four were *D. variabilis* (one nymph and three adults). We found 18 *A. americanum* (55.5%) and one *D. variabilis* (25.0%) were positive for *R. amblyommatis,* while one *A. americanum* (3.0%) was positive for *Rickettsia buchneri*.

### Tick phenology

**Fig 2:**
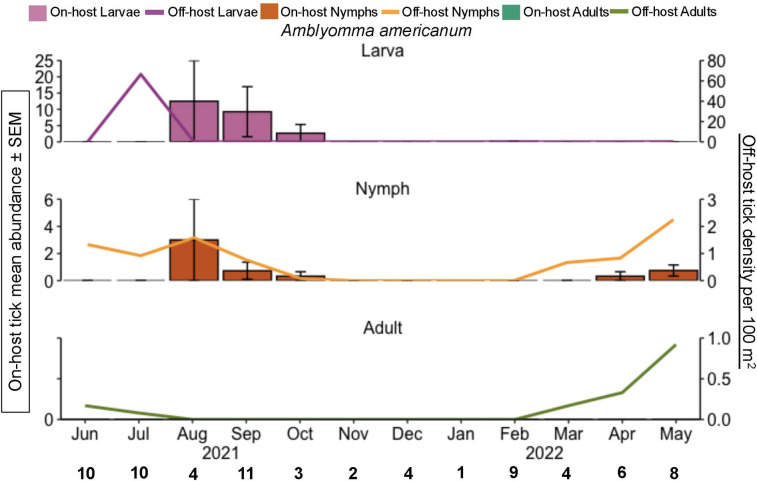
*A. americanum* phenology of both on-host and off-host ticks during June 2021 through May 2022, east Texas, USA. The primary y-axis is the on-host tick mean abundance per month for all hosts sampled (bar graphs). Error bars represent the standard error of the mean. The bold black numbers along the x-axis are the number of mammals checked for ticks each month. The secondary y-axis is host-seeking ticks per 100 m^2^ (line graph). Pink = larvae; orange = nymphs, green = adults.

Off-host density of *A. americanum* adults peaked in the spring (Fig 2), which is also when they were removed from humans the most. A total of 23 adult *A. americanum* ticks were collected in the spring months from both off- hosts and on humans. Off-host *A. americanum* nymphal density followed a bimodal pattern where they were most abundant in the summer (46 ticks) but also found throughout spring and fall. Mirroring this pattern, on-host *A. americanum* nymphs were most abundant in late summer (12 ticks) and into the fall, then again a small abundance on wildlife in the spring (Fig 2). Off-host larval *A. americanum* abundance peaked in the summer (798 ticks; Fig 2), followed by a peak of on-host larvae in the summer and fall (160 ticks).

**Fig 3:**
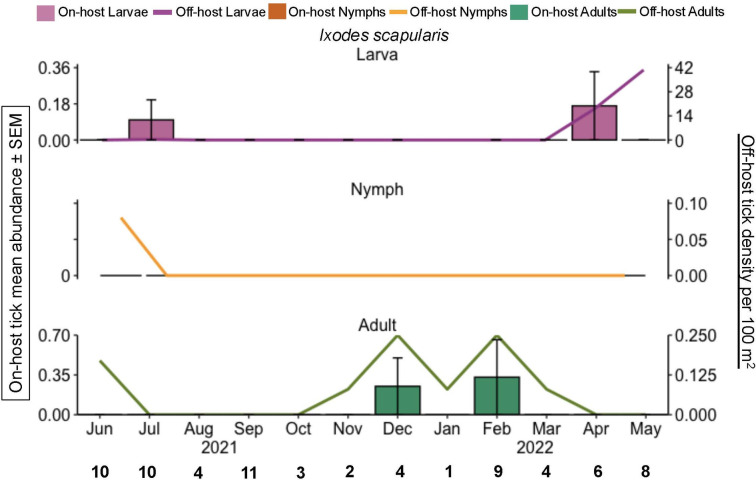
*I. scapularis* phenology of on-host and off-host ticks during June 2021 through May 2022, east Texas, USA. The primary y-axis is the on-host tick mean abundance per month for all hosts sampled (bar graphs). Error bars represent the standard error of the mean. The bold black numbers along the x-axis are the number of mammals checked for ticks each month. The secondary y-axis is host-seeking ticks per 100 m^2^ (line graph). Pink = larvae; orange = nymphs, green = adults.

For adult *I. scapularis*, both off and on-host peaked in abundance during the winter (Fig 3), with 4 ticks removed from hosts and 8 ticks off-hosts. Only a single *I. scapularis* nymph was found in this study; this individual was collected off-host via drag sampling in June (Fig 3). Off-host larval *I. scapularis* abundance peaked in the spring (708 ticks) but were sparse on hosts (2 ticks, Fig 3).

**Fig 4:**
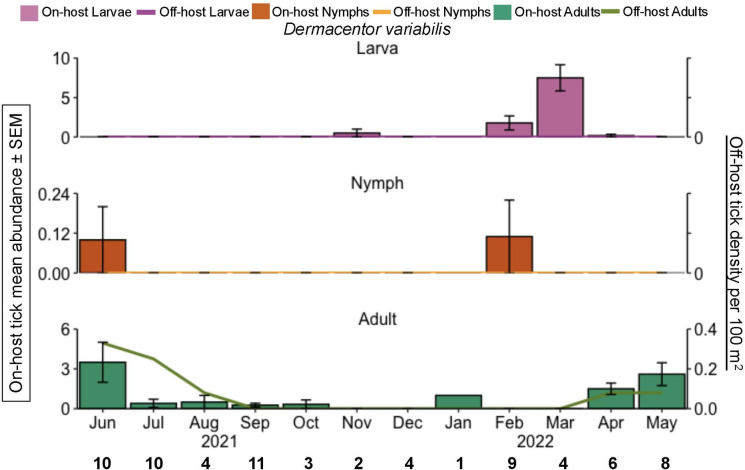
*D. variabilis* phenology of on-host and off-host ticks during June 2021 through May 2022, east Texas, USA. The primary y-axis is the on-host tick mean abundance per month for all hosts sampled (bar graphs). Error bars represent the standard error of the mean. The bold black numbers along the x-axis are the number of mammals checked for ticks each month. The secondary y-axis is host-seeking ticks per 100 m^2^ (line graph). Pink = larvae; orange = nymphs, green = adults.

Increased off-host adult *D. variabilis* abundance was found in the summer months (10 ticks), and no other life stages were collected via drag sampling (Fig 4). Regarding on-host *D. variabilis* increased abundance, adults were in spring (30 ticks) and summer (41 ticks), while larva were in winter (16 ticks) and spring (31 ticks).

**Fig 5:**
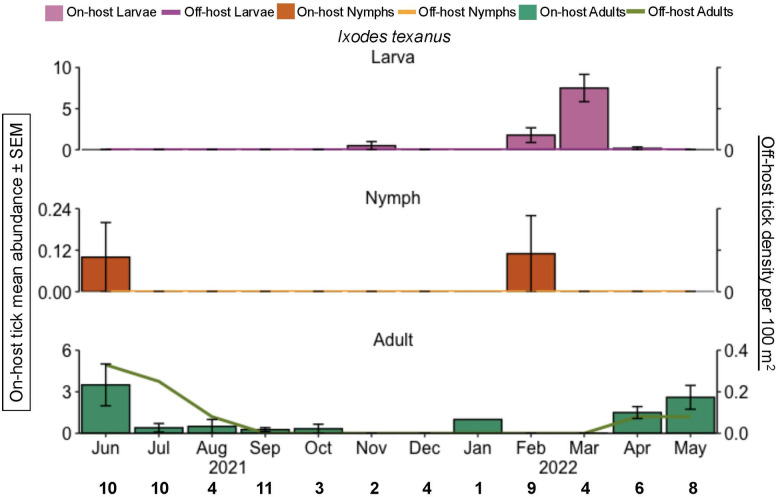
*I. texanus* phenology of on-host ticks during June 2021 through May 2022, east Texas, USA. The y-axis is the on-host tick mean abundance per month for all hosts sampled (bar graphs). Error bars represent the standard error of the mean. The bold black numbers along the x-axis are the number of mammals checked for ticks each month. Pink = larvae; orange = nymphs, green = adults.

All life stages of *I. texanus* (commonly called the raccoon tick) were exclusively found on raccoons, with peak abundance of adults in spring (54 ticks) and winter for nymphs (15 ticks; Fig 5).

### *Rickettsia* species phenology

Because we identified *Rickettsia* spp. in all tick species and almost all life stages collected in the standardized sampling of this study, we also found it all year round. Even though the most ticks were collected in summer months (970 ticks) we found only a 23.1% (36/156) prevalence of *Rickettsia* species. Spring had a prevalence of 49.5% (138/279) *Rickettsia* spp. among all ticks collected in addition to the second highest abundance (919 ticks). Fall had the second highest *Rickettsia* spp. infection prevalence of 44.9% (22/49) but we only collected 140 ticks from on and off-hosts. Lastly, in the winter we collected the least number of ticks (48 ticks) but identified the third highest *Rickettsia* spp. prevalence of 31.0% (13/42).

The highest abundance of off-host *I. scapularis* larvae (718 ticks) also had the highest number of *R. tillamookensis* positives (27 ticks) in the spring. Only one other off-host tick was positive for *R. tillamookensis*, an *I. scapularis* adult in the winter. Among off-host *A. americanum* adults and nymphs, there were 28 positives in summer, 18 positives in the spring, and 6 positives in the fall. No *R. amblyommatis* positives were identified in off-host larval *A. americanum* or off-host ticks found in the winter. *Rickettsia amblyommatis* was isolated from wildlife hosts in late summer (August), fall (September), and spring (April and May) (Table 1), corresponding to when on-host juvenile *A. americanum* were abundant on hosts (Fig 2).

### Tick- host associations from all wildlife sampled in the study

**Fig 6:**
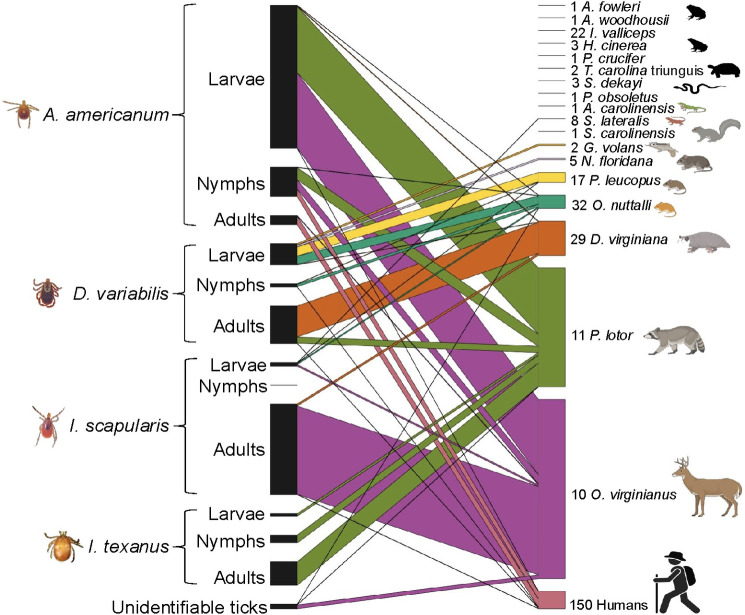
Tick-host associations among all hosts checked for ticks May 2021 to May 2022, east Texas, USA. Numbers next to hosts represent the number of host captures evaluated for ticks. The thickness of each diagonal line corresponds to the number of observations of each tick-host association. Images created in BioRender.

In general, some host species had overall higher tick abundances than others (Fig 6). We collected a total of 427 ticks from ten deer, each having at least one tick but abundances ranged from two to 219 and a mean tick abundance of 42.4 ticks. Across 11 captures of raccoons, we collected 284 ticks ranging from zero to 86 with a mean tick abundance of 25.8 ticks per raccoon. From 29 opossums we collected 82 ticks ranging from 0 to 11 with a mean tick abundance of 2.83 ticks per opossum. The golden mice had slightly more ticks removed (31) than the white-footed mice (24), however mean tick abundances were higher on the white-footed mice (1.41) than the golden mice (0.97). Woodrats and flying squirrels were less abundant in captures and had only four ticks removed from each species. A single *I. scapularis* larvae was removed from a little brown skink in May, averaging to 0.13 ticks per little brown skink and no other herpetofauna captured had ticks present.

Among the tick-host associations (Fig 6), we removed *A. americanum* adults (20 ticks) and nymphs (12 ticks) the most from humans. Additionally a relatively high abundance of *A. americanum* nymphs (51 ticks) and larva (338 ticks) were removed from deer and raccoons.

*Ixodes scapularis* adults were mostly removed from the 10 deer we sampled in November (219 ticks) and only five were collected from three different opossums from the rest of the year (Fig 6). Absolutely no *I. scapularis* nymphs were found on the 150 wildlife hosts checked for ticks or the personnel conducting the field collections. Ten *I. scapularis* larvae were collected from a little brown skink, a white-footed mouse, a deer, and two golden mice (Fig 6). *Ixodes texanus* were only removed from racoons and never found off-hosts, recovering 56 adults, 19 nymphs, and 6 larvae (Fig 6).

Adult *D. variabilis* were mostly removed from raccoons and opossums (90 ticks), while only three were found on humans (Fig 6). Nymphal *D. variabilis* were removed from golden mice (7 ticks), a white-footed mouse (1 tick), and a human (1 tick). Larval *D. variabilis* had the most diverse host range (Fig 6) including white-footed mice (23 ticks), golden mice (20 ticks), a southern flying squirrel (4 ticks), an eastern woodrat (4 ticks), and an opossum (1 tick).

## Discussion

Longitudinal sampling of wildlife and vegetation for a year allowed us to identify seasonal phenology of on and off- host ticks, tick-host associations, and tick-borne bacterial infections of ticks and hosts in a pine-dominated forest of East TX, US. As expected, we found tick peak abundance on and off-hosts varied between tick species and life stage. However, we recovered 98.3% (1,632/1,661) of the off-host ticks in the spring and summer months for all tick species (*A. americanum*, *D. variabilis,* and *I. scapularis*) and life stages; suggesting that there is a heightened acarological risk for people spending time outdoors during these seasons in Texas. As mirrored in the literature [41–43] our data show that larger hosts have higher ectoparasite abundances (Fig 6). We removed only 66 ticks from 57 rodent captures and 711 ticks from just 11 deer and 11 raccoons over the year, indicating that targeting only rodents for integrated pest management would be less efficient that targeting larger animals or multiple hosts [44]. In this narrow spatial scale, a diversity of *Rickettsia* spp. was present within both off-host and on-host ticks across all life stages (Table 2), highlighting the need for more surveillance to describe tick-borne bacterial species in this region at a broader scale.

Mirroring the phenology of *A. americanum* in Oklahoma, we found that off-host adults and larvae peaked in abundance during the same months, May and July respectively [45]. However, we found a longer and earlier peak in activity for nymphal *A. americanum* in Texas [45]. In Oklahoma and Kansas, off-host adult *D. variabilis* were found to be most active in spring months [46,47]. In contrast, we found peak abundance was in June and July (Fig 4). Although full-year phenology study of off-host *I. scapularis* has rarely described in the southern US [48,49], our data show the presence of questing larvae in April and July; a single questing nymph in July, and the presence of questing adults in the winter months (December, February; Fig 3).

*Amblyomma americanum* was the most abundant tick collected both on and off-hosts encountering humans, deer, and raccoons most often (Fig 6). Across all life stages, *A. americanum* was found to have the highest infection prevalence of *R. amblyommatis* both on and off hosts (Table 2). *Rickettsia amblyommatis* was also identified within raccoons while feeding a high abundance of *A. americanum* larvae; we therefore suggest raccoons be assessed further as a primary wildlife reservoir for *R. amblyommatis.* Accumulating evidence indicates that *R. amblyommatis* is a human pathogenic species [19]. The literature lacks investigation on this bacterial species among wildlife and therefore pathogenicity among animals and the capacity of *R. amblyommatis* being zoonotic is still unknown. Our data indicates that public health investigations may therefore benefit from similar multi-season, multi-host sampling efforts across different regions to identify highest risk time periods for infected tick activity.

*Rickettsia tillamookensis* is a relatively newly identified bacteria initially isolated from a patient in the northwest [21] and since has been found in off-host *Ixodes pacificus* nymphs and adults [21] and in off-host *I. scapularis* nymphs and adults [22]. Here, we are the first to describe *R. tillamookensis* in off-host larval *I. scapularis.* This observation allows exploration of the possibility that this species may be vertically transmitted- as is the case for other *Rickettsia* species- because these off-host larvae have not yet engaged in bloodfeeding and thus had no prior opportunity to acquire the infection from a vertebrate host. Furthermore, we detected *R. tillamookensis* from adult *I. scapularis* removed from deer and opossums, in addition to a larvae *I. scapularis* removed from a golden mouse that was positive for *R. amblyommatis*. Due to the sheer abundance of adult *I. scapularis* that feed on deer (Fig 6), we suggest deer be investigated further to see if they may serve as a wildlife reservoir for this agent. These data emphasize the need for an increase in testing *Ixodes* species with *Rickettsia* targeted PCR and sequencing methods so we can learn more about this new *Rickettsia* species.

The species *Rickettsia buchneri* (formerly *Rickettsia* endosymbiont of *I. scapularis* [40]) was the most abundant species identified in ticks, predominantly in *I. scapularis*. Unexpectedly, we also detected this agent in a small number of *A. americanum* and *D. variabilis*. Most ticks we considered positive for this endosymbiont were sequenced across multiple genes, with 100% matches to *Rickettsia monacensis* on the *gltA* assay. This *Rickettsia* species is a causative agent of Mediterranean spotted fever and endemic within *Ixodes* species of Europe [50]. However, it has been recently documented in *Ixodes* spp. removed from dogs in Costa Rica and Nicaragua [51], in ticks from deer in Mexico [52], in *I. scapularis* in Georgia and Florida, USA, [53] and from an *Ixodes* tick removed from a migrating bird in TX, USA [54]. Since rickettsiosis is argued to be a neglected tropical disease and the genus is responsible for most of the emerging vector-borne diseases globally [55,56], better molecular characterization and testing methods are necessary. We utilized a rigorous approach with three different gene targets for testing of all samples, yielding varying results among the different assays; such an approach may not be accessible in routine surveillance or clinical diagnostics and a streamlined and standardized process is warranted.

We identified a hard tick relapsing fever *Borrelia* spp. within a single *D. variabilis* male (Table 2) removed from an opossum in June. The sample was tested with the 16S-23S rRNA IGS nested PCR multiple times resulting in sequences matching to the HT31 strain of *Borrelia miyamotoi*. However, Candidatus *Borrelia texasensis* which was first identified from a *D. variabilis* removed from a coyote in Webb County, TX [57] is another *Borrelia* species within the hard tick relapsing fever group *Borrelia* and does not have a sequence in GenBank representative at this sequence that we amplified. Due to lack of remaining sample, we cannot test our sample to amplify other markers and therefore report it as positive only for a relapsing fever *Borrelia*.

We found no evidence to support local risk of Lyme disease in the study region given our results: 1) we found no ticks or hosts infected with *B. burgdorferi;* 2) we found only a single off-host *I. scapularis* nymph- likely reflecting behavioral difference in which these southern nymphs don’t quest high on the vegetation [48,58,59], and 3) no nymphs were found on any of the 193 hosts we checked for ticks. Similarly, prior studies in Texas report field data suggesting low or no risk [23,24,26]. The role of reptiles- a refractory host for *B. burgdorferi*- in feeding juvenile *I. scapularis* has been hypothesized to contribute to the lower prevalence of *B. burgdorferi* in the southern USA [10,60,61]. Our limited sampling of herpetofauna resulted in finding one larval *I. scapularis* attached to a little brown skink in May (Fig 6). A prior study of 150 skinks from August 2018 to March 2019 in the same county as our study found no attached ticks [62]. Future studies of herpetofauna during periods of juvenile ticks host-seeking phenology will be useful in further understanding of Lyme disease ecology in the region.

Over the full year, trapping success in our study was low (1.2%). In comparison, a previous study in 2015 at the same forest had a trapping success of 13.5% with trapping focused in spring months [26]. The latter study also reported a different rodent community diversity, with the capture of species not represented in our study including *Reithrodontomys fulvescens* (fulvous harvest mouse), *Sigmodon hispidus* (hispid cotton rat), and a *Microtus* spp. (meadow voles). Furthermore, the on-host tick abundances differed, where in 2015 there was an average of 0.75 (79 ticks/106 captured rodents) ticks per rodent compared to in 2021–2022 we found an average of 1.11 (63 ticks/57 captured rodents). We speculate that the climatic anomaly of Winter Storm Uri in February of 2021- during which record amounts of snow and frigid temperatures impacted the state of Texas, resulting in some wildlife die-offs- could have altered the rodent community [63]. Further, rodent populations are known to be cyclic following a boom and bust with resource availability and our observed reduced rodent population could reflect a low point in this cycle. Our work highlights the value of longitudinal surveillance of arthropod and wildlife communities especially in the face of climate change and associated extreme weather events.

Characterizing the foundational tick ecology metrics such as tick phenology, tick-host associations, and tick-borne pathogen prevalence are resource intensive; however, these data are critical for prioritizing public health messaging for tick prevention. Future work should continue standardized monitoring of tick populations and tick-borne pathogens both on and off-hosts, with emphasis on hosts with large home ranges (i.e., birds and large ungulates) and over a larger temporal scale. In the case of our study site, tick exposures may happen year-round, with several *Rickettsia* species present in multiple tick species. Further, our use of molecular approaches to characterize multiple species of tick-borne pathogens within two focal genera (*Rickettsia* and *Borrelia*) revealed a diverse community of *Rickettsia* spp. that may pose risks to humans, and these broad approaches may be increasingly useful especially as presumed endosymbiont species are recognized to be associated with disease.
